# Quality of life in a sample of schoolchildren with attention deficit and hyperactivity disorder

**DOI:** 10.1192/j.eurpsy.2021.617

**Published:** 2021-08-13

**Authors:** S. Pérez Sánchez, I. Martín Herrero, M.A. Cutillas Fernández, D. Raya Güimil, A. Crespo Portero

**Affiliations:** Servicio De Psiquiatría, Servicio Murciano de Salud, Murcia, Spain

**Keywords:** ADHD, schoolchildren, quality of life, methylphenidate

## Abstract

**Introduction:**

Schoolchildren with ADHD have difficulties in different areas of their lives and sometimes need drug treatment. To comprehensively assess the response to treatment, it is interesting to use quality of life questionnaires where the child’s perspective is assessed.

**Objectives:**

To evaluate the quality of life in children with ADHD.

**Methods:**

Sample of 14 schoolchildren from 11 to 14 years of age who attended a primary care check-up and were diagnosed with ADHD under treatment with long-acting methylphenidate. Parent informed consent. AUQUEI questionnaire Spanish versión

**Results:**

Participants answered the questionnaire before starting treatment, at 3 months and 6 months. Four factors were differentiated with different scores: In the baseline results (before treatment), great difficulties were observed in academic performance in 90% (F4, mean 5), family life in 70% (F1, mean 5) and 30% % in leisure (F2, mean 10). After months of treatment, an improvement was observed in the scores regarding academic performance (F4, mean 13) and family life (F1, mean 9). The female sex presented better total scores in quality of life at six months evaluation.

**Conclusions:**

The AUQUEI is an easy-to-apply questionnaire specific to the child population that provides us with a profile from the child’s point of view and can be very useful in the primary care consultation in the comprehensive assessment of the quality of life of the schoolchild with ADHD and pharmacological approach.

